# Our Medical College

**Published:** 1869-04

**Authors:** 


### OUR MEDICAL COLLEGE.

The College of Physicians and Surgeons at Keokuk held its commencement exercises in the Medical College Hall on the evening of February 25th, 1869. The class numbered over one hundred students, and the degree of Doctor of Medicine was conferred upon the graduating class, whose names, residences, etc., we herewith present. The Hall was crowded to its utmost capacity and many citizens and friends of the Institute were compelled to leave for want of room. The order of exercises was as follows:

Music; Prayer; Music; Conferring of Degrees, by the President, Prof. Gillett, M. D. D.D.; Music; Valedictory Address by Prof. J. C. Hughes, M. D.; Announcements by the Dean.

| NAMES OF GRADUATES. | RESIDENCE. | SUBJECT OF THESIS. |
|---|---|---|
| J. E. Alvord | Denmark, Iowa | The Blood. |
| William H. Axline | Fairfield, Iowa | The Delusion of Medicine. |
| Hubert Bethnod | Lyons, France | Gun Shot Wounds. |
| J. W. Carlton | Bentley, Ills | The Blood. |
| H. M. Craton | Jesup, Iowa | Gelseminum. |
| H. C. H. Fitzgerold | Adel, Iowa | Hernia. |
| L. J. Forney | St. Charles, Iowa | Concussion of the Brain. |
| W. F. Gay | New London, Mo | Pneumonia. |
| J. W. Holiday | Decatur, Ills | Terchloride of Formyl. |
| H. C. Heiser | Keokuk, Iowa | Anatomy in Relation to Surgery. |
| Walter T. Hall | Toulon, Ills | Requirements of a Physician. |
| W. F. Hannan | Denver, Ills | Typhoid Fever. |
| Warren Hippee, A. M. | Canton, Ohio | Spermatorrhoea. |
| W. T. Hobbs | Denver, Ills | Malerial Fever. |
| J. A. Horn | Louisiana, Mo | Causes of Disease. |
| J. L. Lemaster | Bushnell, Ills | Amenorrhoea. |
| J. M. McClean | Dallas, Ills | Intermittent Fever. |
| Thomas D. Pollock | Brighton, Iowa | Dyspepsia. |
| Robert H. Reed | Fountain Green, Ills | Human Anatomy. |
| M. Rogers | Carrollton, Mo | Digestion. |
| John J. Reaburn | Denver, Ills | Typhoid Fever. |
| Geo. W. Robinson | Big Mound, Iowa | Obstetrics. |
| C. Snook | Fairfield, Iowa | Coagula in the Blood |
| J. D. Todd | Fayette, Mo | The Pulse in Disease and in Health. |
| C. L. Tate | Farmington, Mo | Physiology of the Digestions. |
| William T. Wright | Bushnell, Ills | Qualifications of the Physician. |

HONORARY DEGREES.—J. B. Brenten, Adel, Iowa; H. E. Hunter, Newton, Iowa.

AD EUNDEM.—Andrew J. Willey, M. D., Newton Iowa.

